# Risk factors and practices contributing to newborn sepsis in a rural district of Eastern Uganda, August 2013: a cross sectional study

**DOI:** 10.1186/s13104-015-1308-4

**Published:** 2015-08-09

**Authors:** Bua John, Mukanga David, Lwanga Mathias, Nabiwemba Elizabeth

**Affiliations:** Department of Health Policy Planning and Management, School of Public Health, Makerere University College of Health Sciences, P.O. Box 7072, Kampala, Uganda; African Field Epidemiology Network (AFENET), P.O BOX 12874, Kampala, Uganda; Kidera Health Centre, Buyende, Uganda; Department of Maternal and Child Health, School of Public Health, Makerere University College of Health Sciences, P.O. Box 7072, Kampala, Uganda

**Keywords:** Newborn care, Newborn Sepsis, Uganda

## Abstract

**Background:**

In Uganda, newborn deaths constituted over 38 % of all infant deaths in 2010. Despite different mitigation interventions over years, the newborn mortality rate is high at 27/1,000 and newborn sepsis contributes to 31 % of that mortality. Therefore, improved strategies that contribute to reduction of newborn sepsis need to be developed and implemented. Understanding the context relevant risk factors that determine and practices contributing to newborn sepsis will inform this process.

**Methodology:**

A cross sectional study was conducted at Kidera Health Centre in Kidera Sub County, Buyende district between January and August 2013. A total of 174 mothers of sick newborns and 8 health workers were interviewed. Main outcome was newborn sepsis confirmed by blood culture. Independent variables included; mothers’ demographics characteristics, maternal care history and newborn care practices. The odds ratios were used to measure associations and Chi square or Fisher’s exact tests to test the associations. 95 % confidence intervals and P values for the odds ratios were determined. Logistic regression was conducted to identify predictor factors for newborn sepsis.

**Results:**

21.8 % (38/174) of newborns had laboratory confirmed sepsis. *Staphylococcus aureus* was the commonest aetiological agent. Mothers not screened and treated for infections during antenatal (OR = 3.37; 95 % CI 1.23–9.22) plus inability of sick newborns to breast feed (OR = 3.9; 95 % CI 1.54–9.75) were factors associated with increased likelihood of having laboratory confirmed sepsis. Women not receiving health education during antenatal about care seeking (OR 2.22; 95 % CI 1.07–4.61) and newborn danger signs (OR 2.26; 95 % CI 1.08–4.71) was associated with laboratory confirmed newborn sepsis. The supply of antibiotics and sundries was inadequate to sufficiently control sepsis within health facility.

**Conclusion:**

Lack of antenatal care or access to it at health facilities was likely to later result in more sick newborns with sepsis. Poor breastfeeding by sick newborns was a marker for serious bacterial infection. Therefore district sensitization programs should encourage women to attend health facility antenatal care where they will receive health education about alternative feeding practices, screening and treatment for infections to prevent spread of infections to newborns. Supply of antibiotics and sundries should be improved to sufficiently control sepsis within the health facility.

## Background

Newborn sepsis contributes to more than 1.6 million deaths annually in developing countries [1] and is therefore an important cause of newborn mortality [2, 3]. The newborn period accounts for 38 % of mortality in children below 5 years and the average daily mortality rate is 30-fold higher than in the post newborn period [4]. Newborn sepsis contributes to 26 % of that newborn mortality [4].

Newborn sepsis occurs when pathogenic bacteria gain access into the blood stream causing overwhelming general infection [5]. The most common isolated organisms causing newborn sepsis in Africa include: *Staphylococcus aureus*, *Escherichia coli*, and *Group B Streptococci* [6, 7]. Infections, prematurity, birth asphyxia, low birth weight and other factors like type of delivery, delivery settings, antenatal care received, newborn mixed feeding and some cultural practices for cord care contribute to incidence of newborn sepsis [4, 8–16]. The lack of laboratory facilities, medical supplies, inequities in service provision, inadequate health care funding are some of the health service factors that contribute to severity of newborn sepsis [17]. Maternal factors that include; febrile illness in a mother with evidence of bacterial infection within 2 weeks prior to delivery, foul smelling liquor, rupture of membranes for more than 24 h and prolonged labour have also been associated with newborn sepsis [10, 18, 19]. Newborns from low socio-economic status and staying in poor environmental conditions have an increased risk of acquiring or developing sepsis [11, 20]. This is because they are exposed to unhygienic postnatal care environment that promotes spread of infection to them [11]. There are also pregnant women who don’t attend antenatal at the health facility therefore missing an opportunity for screening and treatment of infections that could be passed onto their newborns [6, 20]. The use of traditional birth attendants and delivering at home has also been associated with higher risk of newborns developing sepsis especially in developing countries [11, 19, 21].

Newborn sepsis accounts for 31 % of the high newborn mortality rate at 26 per 1,000 live births in Uganda [22]. In Buyende district, newborn sepsis contributes 25 % of newborn deaths, while the newborn mortality rate based on health facility data is 75 per 1,000 live births according to the district health report for 2011. However this could be much higher as more cases occur at home where they die and go unregistered.

The government of Uganda has employed different mitigating interventions including safe delivery campaigns and health education about newborn care over a decade, but progress has been slow in reducing newborn sepsis and other factors contributing to the high newborn mortality rate [22]. Therefore improved prevention, early case detection and management of sepsis strategies are needed. However our knowledge on the specific risks factors that determine and practices contributing to the high newborn sepsis from a population who all present with neonatal illness in rural districts like Buyende District and understanding of the context driving them is limited as there is no documented evidence to explain this.

In order to design appropriate interventions there is need for sound evidence. Therefore this study sought to identify possible context relevant risk factors and how prevalent they were in Kidera County, Buyende district. It also assessed care practices present in the health facilities and households that contributed to newborn sepsis in order to make recommendations that would influence behaviour change at community level.

## Methods

### Study design

This was a cross-sectional study conducted at Kidera Health centre between January and August 2013. Kidera Health Centre is a level four facility located in Kidera County, Buyende District Eastern Uganda. It is the main referral unit for Buyende District. The Health Centre serves the five counties in the district with an estimated population of 248,000 people. The study population was mother and sick newborn pairs admitted at the health facility during the study period. The sick newborns were those admitted with signs and symptoms of sepsis. The definition of neonatal sepsis was adopted from the International Paediatric Sepsis Consensus criteria (PSC) and the Intensive care chapter of Indian Academy of Paediatrics (IAP) [23, 24]. We excluded cases where mothers or newborns were too ill to participate because they had to be referred to Kamuli district hospital for emergency medical care.

### Sample size

The estimated sample size using the formula by Kish Leslie (1965), assuming a prevalence of sick newborns with sepsis to be 37 % [25] and a maximum error of 5 % within a 95 % confidence interval was 183 mother and sick newborn pairs. The level of significance was set at p < 0.05.

### Sampling procedure

All sick newborns who presented to the health facility were screened for signs and symptoms of sepsis by the health workers. The newborns that fitted the selection criteria for having signs or symptoms of sepsis were moved to the admission ward where the health workers first provided routine investigations and provided treatment according to the national guidelines. After the health workers had provided the treatment, the study team sought informed written consent from the mothers to take part in the study. One of the routine investigations was blood culture to confirm sepsis and determine the aetiological agent.

Qualitative data were gathered using a key informant interview question guide for the eight health service providers. They were purposefully selected from the health facility department providing newborn care services and district health team. These included the midwives, nurses and clinical officers at the health facility plus the district focal person for maternal and child health activities.

### Collection and processing of samples procedure

The standard diagnostic test for newborn sepsis in this study was a positive blood culture according to World Health Organization standard guidelines [26]. Blood cultures have a sensitivity that ranges between 80 and 90 % [27, 28]. However in Uganda, most diagnoses for newborn sepsis at lower level facilities (Health Centres) depend on clinical signs and symptoms alone, which results in misclassification of cases [17]. This study used clinical examination to identify the cases and blood culture investigations to confirm the sepsis cases at a Health Centre. The blood cultures identified the aetiological agents while the drug sensitivity tests were done to help determine the appropriate antibiotic treatment regimens to use. The blood samples were collected and processed by a trained district laboratory technician at the health facility. The study provided commercially prepared blood cultures, drug sensitivity discs and an incubator so that the investigations could be done at the health facility. The study used a BBL Septi-Chek manual blood culture system from Dicfo Laboratories.

Approximately 2 ml venous blood was obtained after thorough cleansing of the patients’ skin for 2 min with povidine iodine and allowing the skin to dry before taking blood. One millilitre of blood was collected in each of two bottles containing brain heart infusion (BHI) in a ratio of blood: BHI of 1:10 and then taken to the laboratory within 30 min. Each bottle was incubated at 35 °C for 24 h following which it was examined for visible growth and Gram stain was done. Subcultures were done on blood, Chocolate and MacConkey agar from those blood culture bottles which showed presence of bacteria on physical examination and Gram stain. The agar plates were incubated under aerobic conditions [29]. The identification of the microbial isolates was by a combination of physical and biochemical methods that included gram stain, catalase reaction, coagulase reaction, colony morphology and haemolytic activity on blood agar [30]. Oxidase and citrate tests were done for identified gram negative isolates [29].

For a blood cultures which showed no visible growth and was negative on Gram stain, three subcultures were done on blood, Chocolate and MacConkey agar and observed for a maximum of 7 days before being discarded as negative if there was no growth. All culture bottles with mixed growths (defined as more than 2 types of bacteria) were discarded.

The disk diffusion method adopted from the Clinical laboratory Institute was used to assess the antimicrobial susceptibility of all the isolates [31]. We used the commonly used antimicrobial agents in the Uganda National treatment guidelines for newborn sepsis to assess the susceptibility of the isolates.

### Study tools

Pre-coded, pre-tested, semi-structured questionnaires were used to collect information from the mothers of newborns. The key informant interview (KI) guide was used to interview health providers. The questionnaires and interview guide were pre-tested by the Principal Investigator at Kamuli district hospital.

### Data collection

The semi-structured questionnaires were administered by four trained research assistants who were supervised by the Principal Investigator. The questionnaires used to interview mothers of newborn were translated into the local language (Lusoga). The interviews were conducted in the local language and immediately transcribed to English. The Principal investigator and research assistants edited the data collected to ensure completeness and consistency. The data were cleaned and stored by the Principal investigator. The Principal Investigator conducted face to face interviews of health providers using the Key Informant interview guide.

The health workers were interviewed to obtain information about challenges in prevention and management of newborn sepsis practices within the facility. The Principal Investigator observed for the availability of equipment and drugs in the facility used in providing appropriate care of newborns.

### Data analysis

The quantitative data collected using the questionnaire were coded, entered, cleaned using Epi data and exported to SPSS version 16 software where additional variables were created for analysis [32]. The primary outcome was a newborn with laboratory confirmed sepsis. The univariate analysis of demographic factors was carried out using frequencies and means to describe the study participants. Bivariate analysis was done to assess the associations between the primary outcome and the independent exposure variables. The independent variables included;

- *Social demographics* such as Age, Education level and Source of income of mother.
- *Antenatal care (ANC) history of mother* such as number of ANC visits attended in last pregnancy, history of being screened for infections, history of receiving health education, Tetanus Toxoid immunisation and history of bacterial infection during pregnancy as well as, treatment received.
- *History of birth circumstances* including place of delivery, type of delivery and type of health provider who assisted in delivery.
- *History of birth outcomes* such as Birth weight and gestational age.
- Postnatal care (PNC) history such as history of illness or complications related to delivery and treatment.
- *Newborn care practices* such as Cord care, feeding practice, and cleaning practice.

The continuous independent variables were categorized and their association with the primary outcome was established using Chi square tests or Fisher’s exact test (for tables where the expected cell values were less than 5). The measure of association for categorical variables was Odds Ratios. The P values as well as 95 % confidence intervals for the Odds Ratios were determined.

Variables found to be significantly associated with laboratory confirmed newborn sepsis at 95 % level in bivariate analysis were included in the multivariate logistic regression analysis using 0.05 for entry and 0.1 for exit P values. Confounding and interaction between the various independent variables was assessed using logistic regression. A stepwise logistic regression model was constructed using a backward elimination approach. Multivariate logistic regression was applied to produce odds ratios of associations between the independent variables and primary outcome of laboratory confirmed sepsis, and adjusted to address the influence of other significant variables. The associations in the multivariate logistic regression analysis that were statistically significant (p > 0.05) were included in the final results.

Qualitative data from key informant interviews were transcribed, coded, analysed and separated into themes. It was triangulated with the findings from the questionnaires to gain a deeper understanding of the information observed.

### Ethical considerations

The ethical approval was obtained from Makerere University School of Public Health Institutional Review Board, the Higher Degrees and Ethics Committee and the National Council of Science and Technology. Buyende District health authorities and in-charge of Kidera health centre were asked for permission to use their facilities. Informed written consent was obtained from all the participants after explaining the risks and benefits of the study before they were interviewed. We used anonymous identifiers on the questionnaires to ensure privacy of the participants. All the members of the study team complied with good clinical practices.

## Results

A total of 174 mother and newborn pairs participated in the study. This was 95 % of the estimated sample size. The 8 key informant interviews were of health workers, health unit in charge and members of the District health team. The mothers interviewed were aged between 16 and 44 years (mean 26, SD 6). The majority (66.1 %; 115/174) of the mothers had attained only primary education (Table 1). The main occupation for majority of mothers was subsistence farming (73 %; 127/174). The newborns were aged between 1 and 27 days (mean 12 days; SD 7). There were more female newborns (59.2 %) than males. There were 38 (21.8 %) newborns confirmed by blood culture to have sepsis.

**Table 1:** Demographic factors of the 174 mothers who participated in the newborn sepsis study in Kidera County, Buyende District 2013

	Frequency (%)
Age group in years	
16–19	20 (11.5)
20–29	102 (58.6)
30–39	46 (26.4)
40+	6 (3.4)
Education level	
None	14 (8)
Primary (1–7)	115 (66.1)
Secondary (1–6)	35 (20.1)
Tertiary	10 (5.7)
Occupation	
Peasant farmer	127 (73)
Other occupations	47 (27)

The commonest organism isolated was *Staphylococcus aureus* (31.6 %), followed by *Neisseria meningitidis* (28.9 %), *Escherichia coli* (13.2 %) and *Streptococcus pyogenes* (10.5 %). The other organisms isolated included *Haemophilus influenzae*, *Salmonella species*, *Klebsiella pneumoniae* and *Streptococcus pneumoniae* (Table 2). No *Group B streptococci* was isolated. There was high microbial resistance to penicillin, but high susceptibility to Gentamicin as shown in Table 3.

**Table 2:** Organisms isolated from newborns with laboratory confirmed sepsis in Kidera County, Buyende District 2013

	Frequency	Percent
*Staphylococcus aureus*	12	31.6
*Neisseria meningitidis*	11	28.9
*Escherichia coli*	5	13.2
*Streptococcus pyogenes*	4	10.5
*Haemophilus influenzae*	2	5.3
*Salmonella species*	2	5.3
*Klebsiella pneumoniae*	1	2.6
*Streptococcus pneumoniae*	1	2.6
Total	38

**Table 3:** Resistance pattern of isolated organisms to antibiotics (numbers and percentages)

Drug	Penicillin	Cloxacillin	Chloramphenicol	Gentamicin
*Staphylococcus aureus n/*12 (%)	12 (100)	0 (0)	5 (41.7)	0 (0)
*Neisseria meningitidis n*/11 (%)	11 (100)	0 (0)	8 (72.7)	0 (0)
*Escherichia coli n*/5 (%)	5 (100)	0 (0)	0 (0)	0 (0)
*Streptococcus pyogenes n*/4 (%)	4 (100)	2 (50)	0 (0)	0 (0)
*Haemophilus influenzae n*/2 (%)	0 (0)	2 (100)	2 (100)	0 (0)
*Salmonella species n*/2 (%)	2 (100)	0 (0)	0 (0)	0 (0)
*Klebsiella pneumoniae n*/1 (%)	1 (100)	0 (0)	0 (0)	0 (0)
*Streptococcus pneumoniae n*/1 (%)	1 (100)	1 (100)	0 (0)	0 (0)

### Demographic factors of the mothers and Newborn sepsis

There was a high proportion of laboratory confirmed newborn sepsis cases among mothers in 20–29 years age group, with only 7 years education and farming occupation (Table 4). Differences among these groups were however not statistically significant (Table 4).

**Table 4:** Demographic factors of the 174 mothers versus newborns with laboratory confirmed sepsis in Kidera County, Buyende District 2013 (bivariate analysis)

	Frequency (n)	Newborns with laboratory confirmed sepsis (%)	Odds ratio	95 % CI	Chi square test (*p* value)
Age group in years					
16–19	20	4 (2.3 %)	1.00		
20–29	102	24 (23.5 %)	1.23	0.375–4.035	1.00
30–39	46	8 (17.4 %)	0.84	0.222–3.199	1.00
40+	6	2 (1.1 %)	2.00	0.265–15.082	0.60
Education level					
None	14	2 (14.3 %)	1.00		
Primary (1–7)	115	28 (24.3 %)	1.93	0.407–9.156	0.52
Secondary (1–6)	35	8 (22.9 %)	1.78	0.327–9.656	0.70
Tertiary	10	0 (0 %)	0.00		
Occupation					
Peasant farmer	127	32 (25.2 %)	1.00		
Other occupations	47	6 (12.8 %)	0.44	0.169–1.117	0.07

### Newborn factors and sepsis

A higher proportion of confirmed sepsis cases were among sick newborns in first week of life, born before term (OR = 1.91; 95 % CI 0.78–4.66), and delivered at the traditional birth attendant (OR = 1.65; 95 % CI 0.63–4.32) (Table 5). These findings were not statistically significant in this study. However interviews of health providers revealed that many pregnant women in the area still use the traditional birth attendants for delivery services where poor hygiene settings predispose the newborns to infections.

**Table 5:** Newborn and maternal factors versus laboratory confirmed sepsis in Kidera County, Buyende District 2013 (bivariate analysis)

	Frequency (n)	Newborns with laboratory confirmed sepsis (%)	Odds ratio	95 % CI	Chi square test (*p* value)
Newborn factors					
Age group in days					
1–7	54	17 (31.5 %)	1		
8–14	69	13 (18.8 %)	0.51	0.22–1.16	0.11
15–21	33	5 (15.2 %)	0.39	0.13–1.18	0.09
22–27	18	3 (16.7 %)	0.44	0.11–1.71	0.36
Gestational age born					
Term	146	29 (19.9 %)	1		
Preterm	28	9 (32.1 %)	1.91	0.78–4.66	0.15
Place of delivery					
Health facility	99	21 (21.2 %)	1		
Home	45	9 (20 %)	0.93	0.39–2.23	0.87
TBA	26	8 (30.8 %)	1.65	0.63–4.32	0.31
Other	4	0 (0 %)	0		
Developed problems after delivery					
No	125	24 (19.2 %)	1		
Yes	49	14 (28.6 %)	1.68	0.78–3.61	0.18
Maternal factors					
Attended ANC					
Yes	153	29 (19 %)	1		
No	21	9 (42.9 %)	3.21	1.24–8.33	0.01*
Attended ANC three times					
Yes	100	19 (19 %)	1		
No	74	19 (25.7 %)	1.47	0.72–3.03	0.29
Reported bacterial infection and received treatment at health facility					
Yes	51	5 (9.8 %)	1		
No	123	33 (26.8 %)	3.37	1.23–9.22	0.02*
Received health education about care seeking					
Yes	100	16 (16 %)	1		
No	74	22 (29.7 %)	2.22	1.07–4.61	0.03*
Received health education about danger signs in pregnancy					
Yes	102	16 (15.7 %)	1		
No	72	22 (30.6 %)	2.37	1.14–4.92	0.02*
Received health education about danger signs after delivery					
Yes	87	13 (14.9 %)	1		
No	87	25 (28.7 %)	2.3	1.08–4.86	0.03*
Received health education about newborn danger signs					
Yes	96	15 (15.6 %)	1		
No	78	23 (29.5 %)	2.26	1.08–4.71	0.03*
Newborn care practices					
Exclusive breast feeding					
No	6	1 (16.7 %)	1		
Yes	168	37 (22 %)	1.41	0.16–12.47	1.00
Clean cord care					
Yes	14	1 (7.1 %)	1		
No	160	37 (23.1 %)	3.91	0.50–30.90	0.31
Hand washing					
No	136	28 (20.6 %)	1		
Yes	38	10 (26.3 %)	1.38	0.60–3.17	0.45
Use separate bathing basin					
Yes	76	11 (14.5 %)	1		
No	98	21 (21.4 %)	1.61	0.72–3.59	0.24

* Statistically significant association.

### Maternal antenatal factors and Newborn sepsis

The odds of sick newborns whose mothers never attended antenatal at the health facility were three times higher than those whose mothers did attend, to have blood culture confirmed sepsis (OR = 3.21; 95 % CI 1.24–8.33) (Table 5).

The odds of sick newborns whose mothers received no treatment for bacterial infection during the antenatal period were three times higher than those whose mothers were treated, to have blood culture confirmed sepsis (OR = 3.37; 95 % CI 1.23–9.22) (Table 5). The mothers who received no health education during their antenatal period about care seeking behaviours (OR = 2.22; 95 % CI 1.07–4.61), danger signs in pregnancy (OR = 2.37; 95 % CI 1.14–4.92), danger signs after delivery (OR = 2.3; 95 % CI 1.08–4.86) and for the newborn (OR = 2.26; 95 % CI 1.08–4.71) had higher odds of their sick newborns having blood culture confirmed sepsis than those who did receive health education (Table 5).

### Newborn care practices and sepsis

A high proportion of confirmed sepsis cases was seen among sick newborns whose mothers practised inappropriate cord care, no strict hand washing before handling the baby and shared bathing utensils with rest of the household (Table 5). However the differences among these groups weren’t statistically significant.

Clean cord care involved using clean water to clean the cord and leaving it to dry naturally. But a midwife at the health facility noted that most mothers were influenced by local traditional practices to apply different remedies to the cord of the newborn thus predisposing them to infection.

### Relationship between clinical symptoms and newborn sepsis

The odds of sick newborns who presented with inability to breastfeed on admission were four times higher than those who didn’t, to have blood culture confirmed sepsis (OR = 3.59; 95 % CI 1.58–8.14) (Table 6). The odds of sick newborns presenting with no diarrhoea symptom were four times higher than those who did, to have confirmed sepsis (OR = 3.73; 95 % CI 1.46–9.52) (Table 6).

**Table 6:** Comparison between clinical symptoms and laboratory confirmed newborn sepsis in Kidera County, Buyende District 2013 (bivariate analysis)

	Frequency (n)	Newborns with laboratory confirmed sepsis (%)	Odds ratio	95 % CI	Chi square test (*p* value)
Inability to breast feed					
No	141	24 (17 %)	1		
Yes	33	14 (42.4 %)	3.59	1.58–8.14	0.001*
Fever					
No	16	6 (37.5 %)	1		
Yes	158	32 (20.3 %)	0.42	0.14–1.25	0.11
Convulsions					
No	149	30 (20.1 %)	1		
Yes	25	8 (32 %)	1.87	0.74–4.74	0.19
Difficulty breathing					
No	121	23 (19 %)	1		
Yes	53	15 (28.3 %)	1.68	0.79–3.56	0.17
Diarrhoea					
Yes	62	6 (9.7 %)	1		
No	112	32 (28.6 %)	3.73	1.46–9.52	0.003*
Skin rash					
No	120	22 (18.3 %)	1		
Yes	54	16 (29.6 %)	1.87	0.89–3.95	0.10
Hypothermia					
No	153	32 (20.9 %)	1		
Yes	21	6 (28.6 %)	1.51	0.54–4.21	0.43

* Statistically significant association.

### Factors associated with blood culture confirmed newborn sepsis at logistic regression analysis

The odds of sick newborns who presented at the clinic with inability to breast feed were four times higher than those who didn’t, to have confirmed sepsis (95 % CI 1.543–9.753) (Table 7). While the odds of sick newborns whose mothers had a history of bacterial infection during antenatal and received treatment (OR = 0.15; CI 0.05–0.45) plus sick newborns who had symptoms of diarrhoea (OR = 0.22; CI 0.08–0.59) were less than those who didn’t, to have confirmed sepsis (Table 7). The age group and level of education of mothers were found to be confounders therefore they were dropped from the model.

**Table 7:** Factors associated with laboratory confirmed newborn sepsis after multivariate logistic regression analysis

Factor	Adjusted odds ratio	95 % CI	P value
Mother with history of bacterial infection and received treatment			
(Base = No)			
Yes	0.151	0.050–0.454	0.001
Inability to breast feed			
(Base = No)			
Yes	3.88	1.543–9.753	0.004
Newborn with diarrhoea			
(Base = No)			
Yes	0.218	0.081–0.586	0.003
−2Log likelihood	152.61		

### Health facility factors

The interviews of the health providers revealed that the health facility didn’t have adequate funding to ensure availability of medical sundries like disinfectants and gloves to use in keeping the maternity ward clean to prevent spread of infection.

The existing infrastructure at the health centre wasn’t adequate enough to cater for pregnant women in labour, women in immediate postnatal and their newborns. This provided an environment for spread of infection.

The handling of mothers during delivery may not be very clean. So infection may be passed on to the newborn. This may happen in cases where a mother ends up delivering on the floor because the two delivery beds we have are already being used by other people. (Key informant, Nurse in Kidera Health Centre).

We have no special room or beds for caring for newborns after delivery. So after delivery they share the same bed with their mothers. (Key informant, Maternity in charge in Kidera Health Centre).

The treatment of sick newborns at the health facility was hindered by inadequate supply of intravenous and syrup antibiotics. This resulted in the health facility experiencing drug stock outs which also discouraged mothers of sick newborns from seeking treatment there.

We don’t receive enough intravenous and syrup antibiotics to treat sick newborns at the health facility. So we usually ask mothers to buy from the local drug shops. This discourages some mothers with sick newborns from coming to the health facility for treatment. (Key informant, Midwife at Kidera Health Centre).

The health facility had no capacity to offer routine screening for bacterial infection of pregnant women during antenatal because it lacked equipment and adequate supply of reagents to do so. However the health facility could offer routine HIV and malaria screening services which were well funded by different district health programmes.

## Discussion

This study revealed that sepsis was laboratory confirmed in 21.8 % of the 174 sick newborns admitted based on clinical signs and symptoms. This was less than the 37 % reported by Mugalu et al. [25] at Mulago National Referral Hospital. The number of laboratory confirmed cases could have been higher because 49.4 % (86/174) of the mothers who participated reported having first sought treatment at a local drug shop or clinic before they came to the health facility for better management.

The commonest aetiological agent was *Staphylococcus aureus* (31.6 %). This was similar to findings of Mugalu et al. and Seale et al. in Sub Saharan Africa [7, 25]. Most of the identified aetiological agents were resistant to penicillin except for *Haemophilus influenzae*. The resistance to penicillin could have been due to inappropriate use of antibiotics for treating sick newborns at home for various ailments before mothers sought care with a qualified health provider. The mothers in this area have easy access to antibiotics from local drug shops. Only *Streptococcus pyogenes*, *Haemophilus influenzae* and *Streptococcus pneumoniae* were resistant to Cloxacillin. There was resistance to Chloramphenicol by *Staphylococcus aureus* (41.7 %), *Neisseria meningitides* (72.7 %) and *Haemophilus influenzae* (100 %). All the identified agents were susceptible to Gentamicin which is used to treat newborn sepsis in lower level health facilities within Uganda. However Gentamicin should be administered in combination with other antibiotics like ampicillin, and cloxacillin to prevented development of new resistant microbial agents.

### Maternal factors

This study revealed no statistically significant difference among the age groups, levels of education and occupation of the mothers. Although evidence from Adejuyigbe et al. [20] in Nigeria showed that mothers from lower socio-economic status that included both low education and earning level had higher odds of experiencing their newborns acquiring sepsis.

The pregnant mothers attending antenatal care at a health facility was found to be beneficial to newborns because they were more likely to be screened and given treatment for infections. This reduced the chances of transmitting infections to their newborns as compared mothers who never attended any antenatal clinic for screening and treatment. This was similar to findings of an assessment made about risk-based antibiotic prophylaxis interventions by Schrag et al. [33] following screening during antenatal.

The health education messages pregnant mothers received during antenatal about care seeking and dangers signs of a newborn were important in increasing early detection of sepsis. That information acquired also influenced mothers to use appropriate care practices during pregnancy and after delivery to minimize the chances of their newborns having sepsis.

### Newborn factors

Chan et al. [34] demonstrated that preterm and the first 7 days of a newborn are associated with a higher chance of acquiring sepsis. But this study demonstrated no statistically significant difference among those groups. Children born before term have a higher likelihood of getting sepsis as compared to those born at term and beyond [35].

The odds of sick newborns who presented with difficult to breastfeed on admission were four times higher than for those who could, to have confirmed sepsis. Poor feeding by sick newborns was an indicator for severe bacterial infection. When a newborn fails to breast feed either due to severity of disease or other cause mothers tend to use other feeding mechanisms which may increase the likelihood of passing on infections. The newborns also miss getting maternal antibodies in the breast milk that can offer them protection against common infections. There was a higher proportion of laboratory confirmed sepsis cases in newborns with no diarrhoea symptom than those who experienced it. This observation could have been due to a common practice of mothers in rural areas first seeking antibiotic treatment for diarrhoea from local drug shops before they visited the health centre.

### Newborn care practices

Mullany et al. [14], Bahl et al. [12], Edmond and Zaidi [11] and Kayange et al. [15] demonstrated that practices like unhygienic cord care and not washing hands before handling newborns could predispose them to sepsis. However this study demonstrated no statistically significant difference among the groups even though a higher proportion of newborn sepsis cases were observed in mothers who practiced hand washing and inappropriate cord care.

### Health facility factors

The evidence by Bhutta et al. [21], Edmond and Zaidi [11] and Ojukwu et al. [19] demonstrated that the newborns delivered at homes or by traditional birth attendants in unsterile settings had a higher likelihood of acquiring sepsis as compared to babies delivered in health facilities were sterility was ensured. This study revealed a higher proportion of confirmed sepsis cases in sick newborns delivered by traditional birth attendants as compared to those delivered in the health facility. Even though that observation wasn’t statistically significant, traditional birth attendant settings usually don’t guarantee sterility and clean delivery practices thus expose newborns to infection.

The insufficient supply of disinfectants, gloves and intravenous antibiotics meant that the health workers can’t sufficiently control the spread of sepsis to newborns within the health facility. The inadequate infrastructure in terms of few delivery beds forced some mothers to give birth on the floor, lack of beds caused mothers to share with their newborns and lack of a treatment room for sick newborns were also problems experienced by health workers in controlling the spread of infection to newborns within the facility. The health facility also lacked laboratory equipment and adequate supply of reagents to provide routine comprehensive infection screening of pregnant women receiving antenatal care at the health facility.

### Study limitations

Since the study was based at the facility, newborns with symptoms of sepsis and weren’t brought to the health facility for medical care could have been missed resulting in low external validity. However we worked with the community health workers who sensitized mothers in the villages within the catchment about symptoms of newborn sepsis and encouraged them to seek care at the health facility. This helped to increase our coverage. The exclusion of mothers or newborns too ill to participate because they had to be moved to a District hospital for emergency medical care created some selection bias. The method used was also unable to establish causal inference for identified local factors and this needs further study.

The study due to limited resources couldn’t perform more advanced tests on bacterial isolates. These included hippurate hydrolysis, CAMP tests, triple sugar iron agar reaction, indole, urease and Voges Proskauer Test. The use of non-automated blood culture methods also reduced culture sensitivity.

## Conclusion

The prevalence of laboratory confirmed newborn sepsis was 21.8 % and *Staphylococcus aureus* was the commonest aetiological agent. The lack of antenatal care or access to it at the health facility was likely to result in more sick newborns with sepsis.

Therefore district sensitization programs should encourage pregnant women to seek antenatal care at the health facilities where they can be health educated, comprehensively screened and treated for infections to prevent spread of infections to newborns. Mothers should also be encouraged to continue exclusive breastfeeding of sick newborns. However alternative clean feeding practices for newborns too sick to breastfeed should also be taught to mothers. Supply of antibiotics and medical sundries should also be strengthened so that health workers can sufficiently control sepsis within the health facility.
